# BrCaM an artificial intelligence model for surgical decision making in breast cancer

**DOI:** 10.1038/s41598-026-43281-6

**Published:** 2026-03-16

**Authors:** Daniela Evangelista, Vasuk Gautam, Luca Silvestri, Mario Zanfardino, Monica Franzese, Massimiliano D’Aiuto

**Affiliations:** 1 Institute of Food Science, Italian National Research Council, via Roma 64, Avellino, 83100 Italy; 2 Norton Neuroscience Research Institute, 3991 Dutchmans Ln Suite 302, Louisville, KY 40207 USA; 3 University of Rome Tor Vergata, Via della Ricerca Scientifica 1, Rome, 00133 Italy; 4 IRCCS SYNLAB SDN, Via G. Ferraris 144, Naples, 80143 Italy; 5 Breast Unit Aziendale, Presidio Ospedaliero di Boscotrecase, ASL Napoli 3 Sud, Naples, Italy

**Keywords:** Breast cancer, Mastectomy, Breast conserving surgery (BCS), Machine learning, Clinical prediction models (CPM), Cancer, Medical research, Oncology

## Abstract

Optimizing surgical decisions in breast cancer is critical. Choosing between mastectomy and breast-conserving surgery (BCS) is complex due to heterogeneous pre-operative clinical factors. We developed BrCaM (*Br*east *Ca*ncer *M*odel), a machine learning–based Clinical Prediction Model designed to analyze pre-operative surgical decision patterns. A dataset of 5100 patients (age range: 18–96 years) treated at a Breast Unit with standardized protocols was used. Surgeon-guided feature selection and an end-to-end machine learning pipeline were implemented. Multiple algorithms were evaluated; AdaBoost performed best using 10-fold cross-validation. BrCaM achieved an overall accuracy of 95% in distinguishing BCS from mastectomy. Feature selection identified clinically meaningful predictors that reflect established criteria influencing surgical decisions. In this retrospective setting, BrCaM captures real-world surgical decision patterns based on clinical factors. These findings support the consistency of current clinical practice and provide a foundation for future prospective validation as a clinical decision-support adjunct.

**Supplementary Information:**

The online version contains supplementary material available at 10.1038/s41598-026-43281-6.

## Introduction

**Fig. 1:**
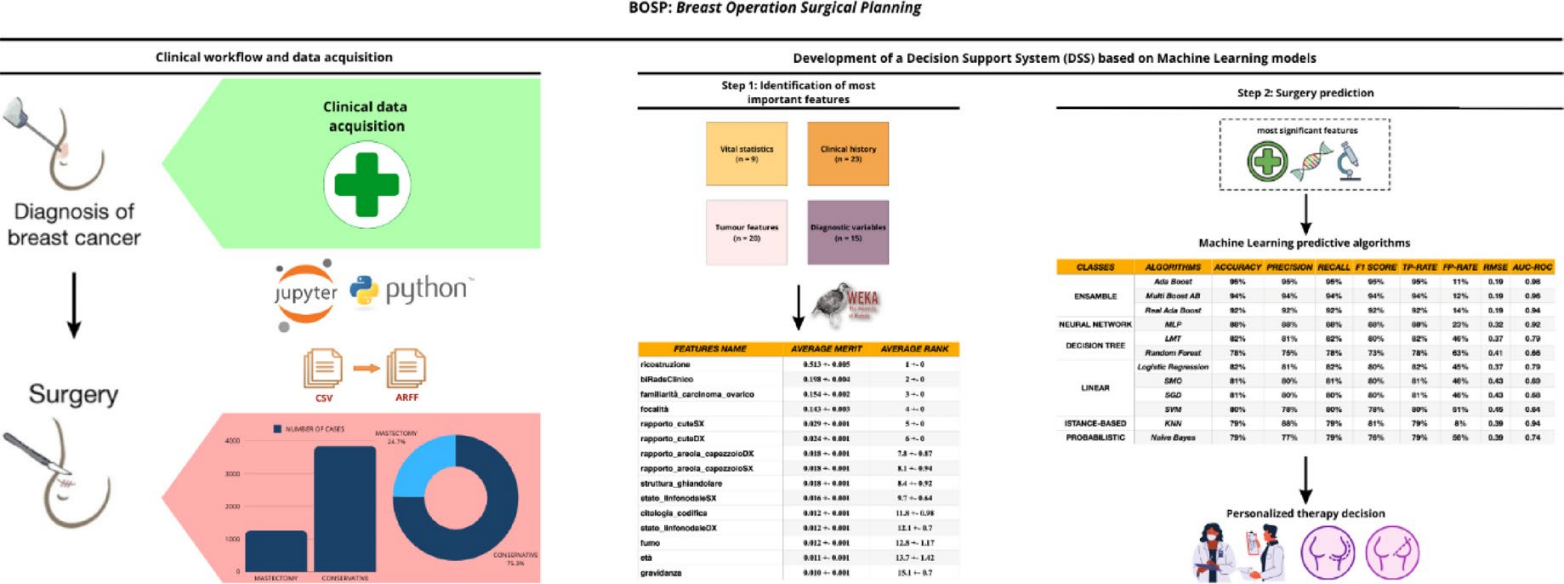
BrCaM system architecture and design. The system integrates clinical data acquisition from hospital databases, processes data through preprocessing and feature engineering pipelines, applies machine learning classification algorithms, and generates personalized surgical recommendations. *N* = 5100 patients, 21 clinical features, AdaBoost classification model with 10-fold cross-validation.

In recent years, artificial intelligence (AI) and machine learning (ML) have played an increasing role in breast cancer management, supporting various stages of treatment, from diagnosis to surgical decision-making. AI models have been developed to assist clinicians in the decision-making process, particularly in determining the most appropriate surgical approach between breast-conserving surgery (BCS) and mastectomy. These models often incorporate tumor characteristics, clinical history, and imaging data to provide personalized treatment recommendations. When appropriately indicated, these procedures provide comparable oncologic safety, yet they differ substantially in terms of functional outcomes, aesthetic results, psychosocial impact, and quality of life^1–4^. Several decision support systems have been proposed in the literature to assist surgical decision-making in breast cancer. Notable systems have proposed AI models for predicting the likelihood of BCS versus mastectomy based on clinical features^5–7^ and who created breast cancer risk models to guide treatment decisions based on patient history and risk factors. While these models focus on risk stratification, they do not directly address the decision between BCS and mastectomy, which is a critical factor influencing both oncologic and quality-of-life outcomes. Other AI systems focus on imaging-based decision support who utilized radiomics to predict tumor characteristics from medical imaging data^8^, which can help inform surgical decisions. Moreover, the integration of multi-omics data in models^9^ offers a more comprehensive understanding of tumor biology, potentially influencing surgical planning by highlighting features that could impact the feasibility of BCS vs. mastectomy. Despite these advances, there remains no dedicated global system that bridges both data containers and machine learning algorithms specifically tailored for surgical planning in breast cancer. Current systems focus on diagnostic assistance, risk assessment, or postoperative outcomes, but few provide real-time decision support to guide pre-operative surgical planning, especially in the context of BCS vs. mastectomy. This study introduces BrCaM (*Br*east *Ca*ncer *M*odel), a machine learning-based framework designed to address this gap by integrating clinical data and predictive modeling to guide surgical decisions. Evidence further suggests that, in selected patients, breast-conserving surgery following neoadjuvant therapy may yield survival and recurrence outcomes comparable or superior to mastectomy^10^. Despite advances in early detection and tailored systemic therapies, breast cancer remains among the most prevalent malignancies worldwide, reinforcing the need for individualized and evidence-based surgical planning^11^. In parallel, recent progress in machine learning (ML) and eXplainable artificial intelligence (XAI)^12–14^ has led to substantial advances in breast cancer research. ML-based models have been applied to risk stratification, survival prediction, treatment response assessment, and postoperative outcome evaluation^15^. For example, explainable ML approaches have been used to compare long-term survival outcomes between mastectomy and BCS, identifying prognostic determinants associated with treatment benefit^16^. Other studies have focused on predicting postoperative quality of life^17^, recurrence risk and survivability^18–20^, and response to neoadjuvant therapy using uncertainty-aware ML frameworks^21,22^. Multimodal deep learning approaches have also been proposed for molecular phenotype inference from imaging data and ultrasound-based breast characterization^23^, while intraoperative AI systems have demonstrated feasibility in supporting robotic breast surgery^24^. Together, these studies highlight the growing translational potential of AI in breast cancer care^25^. Despite this progress, several important gaps remain insufficiently addressed. First, most existing AI applications focus on diagnosis, imaging interpretation, survival analysis, or postoperative outcomes, whereas relatively few studies aim to support pre-operative surgical decision-making, particularly the choice between BCS and mastectomy—an endpoint that directly shapes both oncologic and quality-of-life outcomes^26,27^. Second, many published models rely on heterogeneous, multi-center retrospective datasets characterized by non-standardized diagnostic workflows, variable imaging protocols, and inconsistent surgical indications, limiting reproducibility and hindering real-world clinical deployment^28,29^. Third, few studies provide reprocessing, and predictive modeling within a unified pipeline, thereby reducing their translational applicability^30^. Finally, existing AI systems often incompletely model the multifactorial determinants of surgical feasibility, such as the combined influence of tumour morphology, anatomical constraints, lymph node status, glandular structure, and reconstruction feasibility, resulting in limited interpretability and clinical actionability. To address these limitations, the present study introduces BrCaM (Breast Cancer Surgical Planning), a machine learning–based predictive framework trained on a large and homogeneous cohort of 5,100 patients treated within a single Breast Unit adopting standardized diagnostic and surgical protocols. The primary contributions of this work are: (i) the development of a clinically curated real-world dataset collected under consistent institutional criteria; (ii) the design of a machine learning model specifically focused on predicting the choice between breast-conserving surgery and mastectomy; (iii) the implementation of an end-to-end, clinically deployable pipeline integrating database architecture, surgeon-guided feature selection, data preprocessing, and predictive modeling; (iv) the identification of clinically meaningful predictors influencing surgical choice; and (v) the demonstration of high predictive performance using an AdaBoost-based classification approach. By capturing complex interactions among tumour characteristics, anatomical features, and patient-specific factors, BrCaM aims to support pre-operative surgical planning, reduce decision uncertainty, and complement clinical judgment through data-driven and interpretable decision support (Fig. 1).

## Methods

**Fig. 2:**
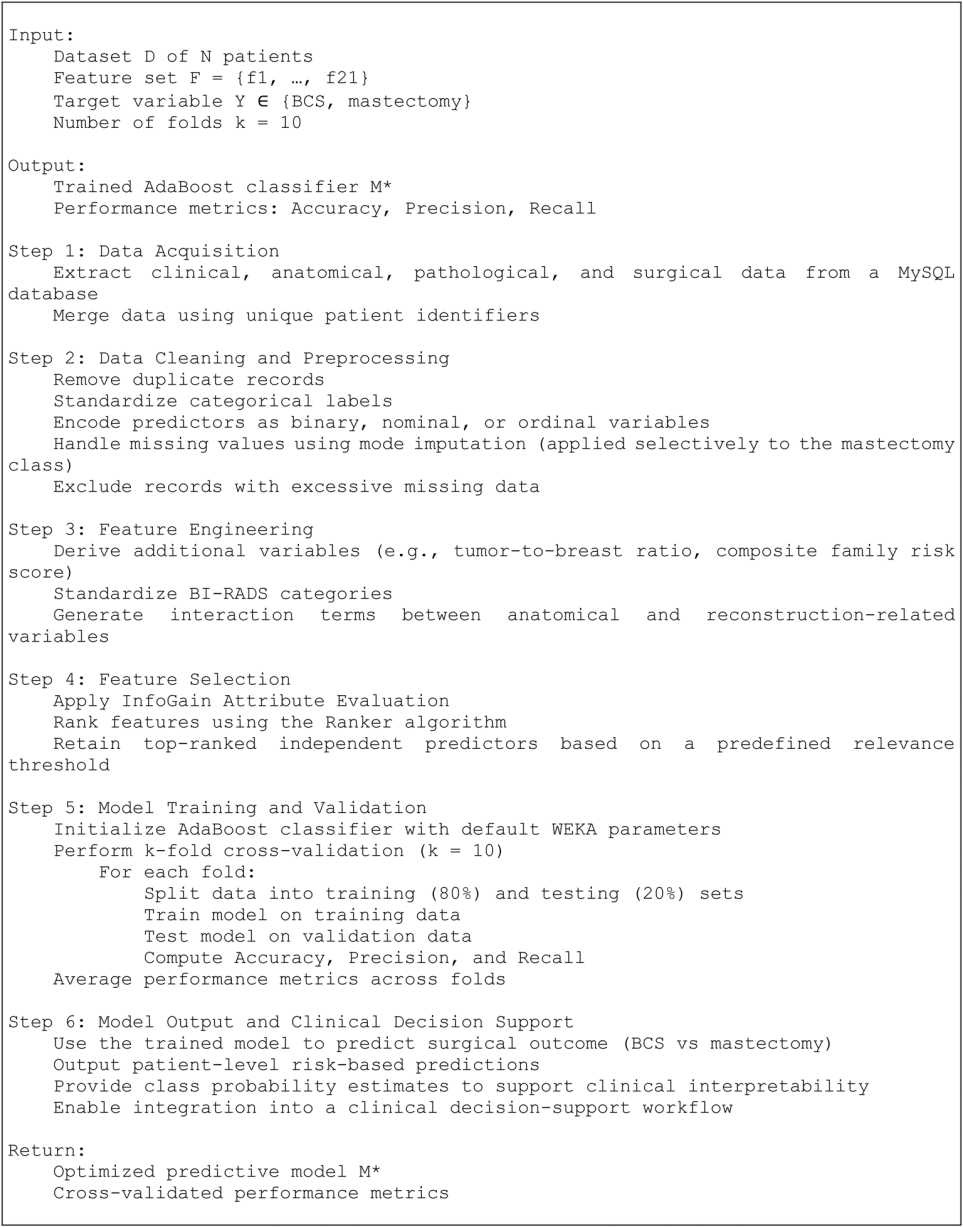
Workflow of the proposed BrCaM predictive model based on AdaBoost. The pipeline consists of six main stages: (1) data acquisition from the relational MySQL database; (2) data cleaning and preprocessing, including encoding and selective mode imputation; (3) feature engineering and label standardization; (4) feature selection using InfoGain and Ranker algorithms; (5) model training and validation through 10-fold cross-validation; and (6) generation of model outputs for individualized surgical decision support between breast-conserving surgery and mastectomy.

An automatic diagnostic and decision-support framework for breast cancer research, prevention, diagnosis, and treatment was developed using supervised classification and prediction techniques. The framework was designed in accordance with the principles of Clinical Prediction Models (CPMs), which estimate patient-specific probabilities of clinical events to support individualized clinical decision-making. Within this framework, the BrCaM (*Br*east *Ca*ncer *M*odel) model functions as a CPM, predicting the probability of breast-conserving surgery (BCS) versus mastectomy based on pre-operative clinical variables. For each patient, BrCaM generates class-specific probability estimates derived from an AdaBoost ensemble, which aggregates weighted weak learners and normalizes their outputs to obtain calibrated probabilities for the two surgical outcomes. A retrospective cohort of 5604 consecutive female patients diagnosed with primary breast cancer between 2009 and 2015 was collected at the Breast Unit of the National Cancer Institute of Naples (IRCCS “Fondazione G. Pascale”). After preprocessing—including duplicate removal, label harmonization, and exclusion of records with extensive missing data—5,100 patients were retained for analysis. Surgical outcome labels were assigned based on operative reports and institutional clinical guidelines, classifying patients into: (i) breast-conserving surgery, defined as preservation of the majority of breast tissue, and (ii) mastectomy, defined as complete breast tissue removal, performed with or without immediate reconstruction. Surgical decision criteria followed standardized protocols incorporating tumor size, multifocality, lymph node status, glandular architecture, skin and nipple–areola involvement, and reconstructive feasibility. The final dataset comprised 3,841 BCS cases (75.3%) and 1,259 mastectomy cases (24.7%), with mastectomy designated as the positive class for model training. A detailed description of all collected variables, including data type and encoding, is provided in the Supplementary Material (Data Dictionary). The BrCaM computational pipeline, illustrated in Fig. 2 and detailed in Algorithm 1, consists of six sequential steps:

*Step 1*, clinical, anatomical, pathological, and surgical data were extracted from a MySQL database and merged using unique patient identifiers to define a cohort of N patients with candidate predictors F = {f_1_, …, f_21_} and a binary outcome Y ∈ {BCS, mastectomy};

*Step 2*, data cleaning and preprocessing were performed, including removal of duplicate records, standardization of categorical labels, variable encoding according to measurement scale (binary, nominal, ordinal), selective mode imputation for missing values where appropriate (including class-specific imputation for mastectomy), and exclusion of records with excessive missingness;

*Step 3*, feature engineering was applied to generate clinically motivated derived variables (e.g., tumor-to-breast ratio and composite family risk score), harmonize BI-RADS categories, and construct interaction terms capturing relationships between anatomical features and reconstruction-related factors;

*Step 4*, feature selection was conducted using an InfoGain attribute evaluator with a Ranker algorithm, retaining predictors exceeding a predefined relevance threshold;

*Step 5*, an AdaBoost classifier was trained and evaluated using 10-fold cross-validation implemented in WEKA, with an 80%/20% train–test split within each fold; model performance was summarized using accuracy, precision, and recall averaged across folds;

*Step 6*, the optimized model (M*) produced patient-level class predictions and probability estimates to support interpretable, risk-informed surgical decision-making.

Performance metrics were reported as cross-validated means to ensure robust estimates of generalizability (Supplementary Materials, Performance.docx). Cohort-level statistics shown in Fig. 6 were generated using WEKA after preprocessing and reflect aggregated, anonymized data, while descriptive statistics reported in Table 1 were computed directly from the same preprocessed dataset, ensuring internal consistency and reproducibility.

### Experimental design

**Fig. 3:**
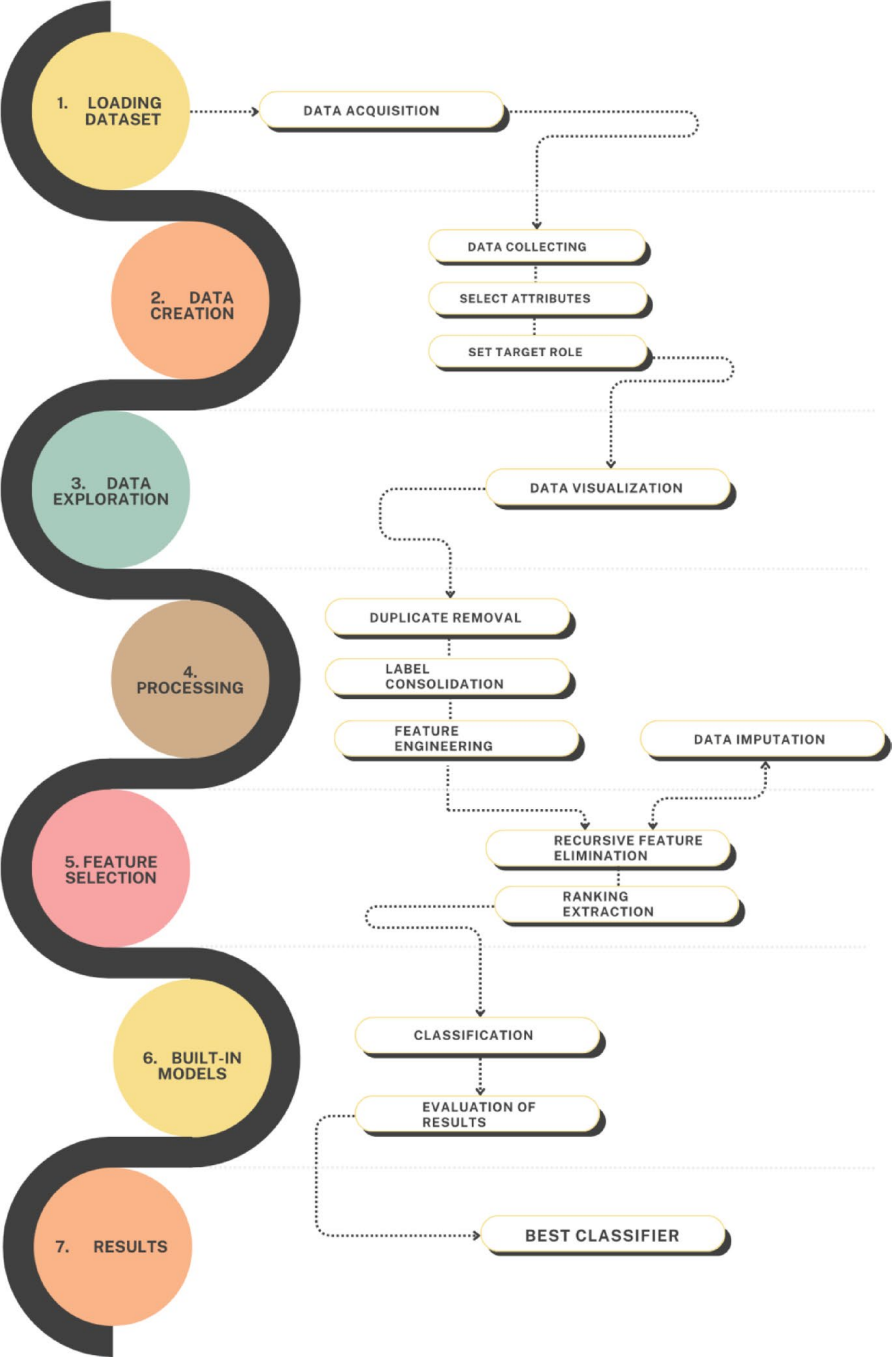
BrCaM computational pipeline workflow. The seven-phase methodology includes: (1) dataset loading from the database (*N* = 5604 initial patients), (2) data creation through clinical data acquisition and attribute selection with target role definition (conservative vs. demolitive surgery), (3) data exploration and visualization, (4) data preprocessing including duplicate removal, label consolidation, feature engineering, and data imputation, (5) feature selection using Info Gain Attribute Eval and Ranker search method to identify final prognostic factors from 64 initial variables, (6) model building and training with multiple classification algorithms (AdaBoost, neural networks, decision trees, linear classifiers, instance-based, and probabilistic methods), and (7) results evaluation through 10-fold cross-validation to identify the best classifier (AdaBoost, 95% accuracy) for surgical planning prediction.

The pipeline (Fig. 3) was developed to manage the full dataset of breast carcinoma patients and to build a predictive model for surgical planning. Its implementation comprised two phases: (i) relational database construction and (ii) predictive model development. Medical records were first collected in a MySQL database^31^, managed through phpMyAdmin^32^. The database stores structured tables including patient demographics, clinical history, tumor characteristics, diagnostic results, anatomical assessment, and surgical outcomes. Data are extracted via SQL queries, preprocessed, and then input to the machine learning pipeline for feature selection and model training (Figs. 1 and 3). The database was organized into tables for: (1) Patient Demographics (age, vital statistics), (2) Clinical History (family history, comorbidities, lifestyle factors), (3) Tumor Characteristics (size, grade, stage, focality, BI-RADS), (4) Diagnostic Results (cytology, imaging, lymph node status), (5) Anatomical Assessment (breast size, glandular structure, nipple-areola measurements), and (6) Surgical Outcomes (procedure type, reconstruction)^33^. Foreign keys maintained data integrity and enabled efficient data extraction for model training.

**Fig. 4:**
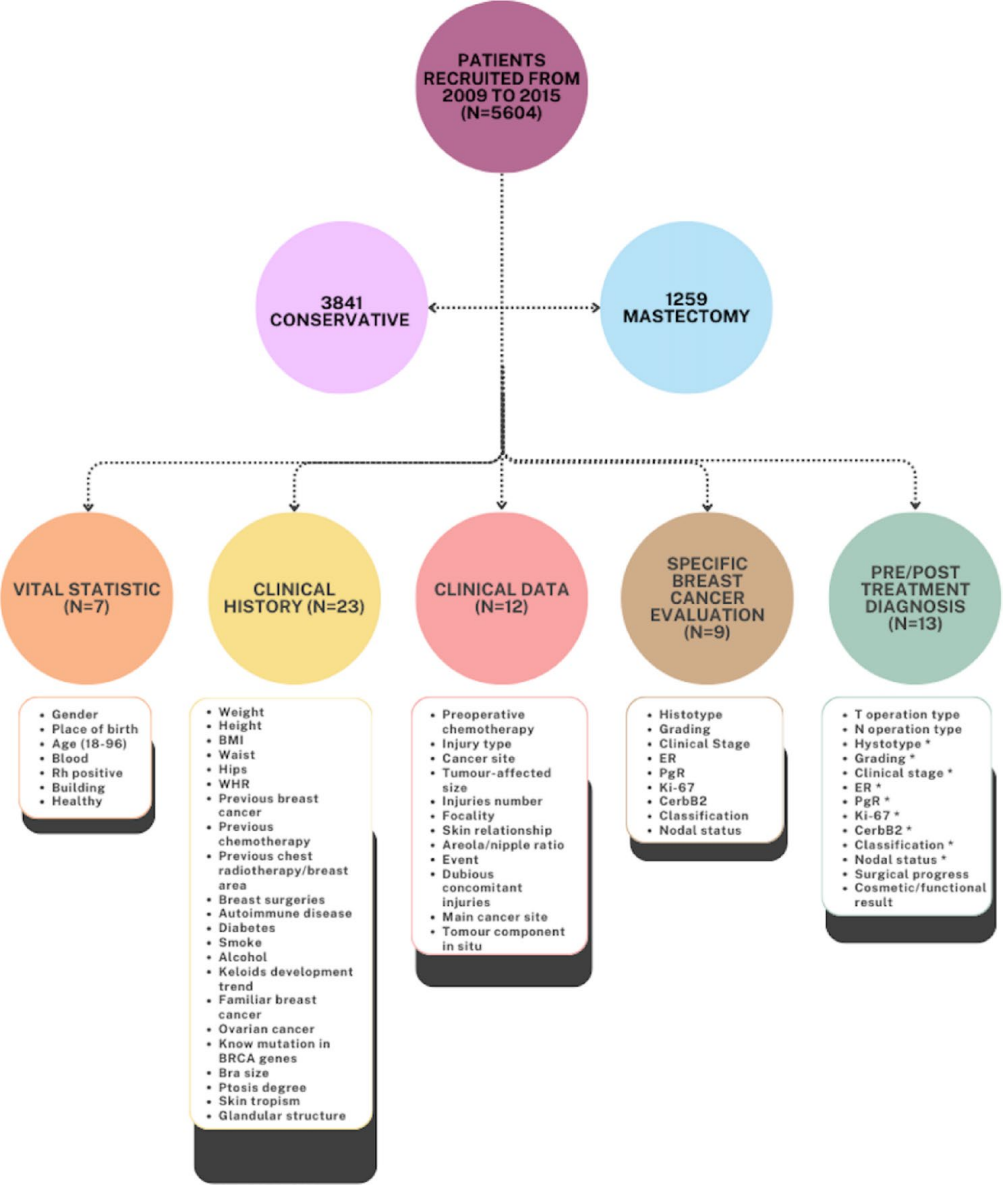
BrCaM dataset structure and composition. Data from 5,604 Italian women (2009–2015) categorized into conservative surgery (*n* = 3,841, 75.3%) and demolitive surgery (*n* = 1259, 24.7%) groups across five feature categories: vital statistics (*N* = 7 variables), clinical history (*N* = 23 variables), clinical data (*N* = 12 variables), breast cancer-specific evaluation (*N* = 9 variables), and pre/post-operative diagnosis (*N* = 13 variables).

After collection, the dataset underwent preprocessing to remove duplicates, standardize labels, and consolidate features. Feature engineering was performed to identify redundant variables, derive new attributes, remove irrelevant features, and eliminate empty variables. Variables considered for patient profiling included vital statistics, clinical history, clinical data, specific breast cancer assessments, and diagnostic parameters. Feature selection identified the most relevant predictors using the Information Gain Attribute Evaluation algorithm and the Ranker search method, producing a subset of 21 prognostic factors. These included age, smoking status, pregnancy history, family history of ovarian carcinoma, glandular structure, nipple-areola ratio, skin relationship, lymph node status, clinical board evaluations, cytology outcomes, tumor focality, and reconstruction feasibility (Fig. 4). Selection was guided by clinical consultation with breast cancer surgeons, ensuring inclusion of factors with established relevance for surgical planning. Missing values were handled with mode imputation applied selectively to the minority mastectomy class, preserving variability and statistical integrity. Feature engineering included tumor-to-breast volume ratios, composite risk scores, standardized categorical labels, age transformations, and interaction terms between reconstruction feasibility and anatomical factors. The predictive framework, BrCaM, functions as a Clinical Prediction Model (CPM), estimating individualized probabilities for conservative versus ablative surgery to guide clinical decisions.

### Computational resources

All analyses were conducted using WEKA (version 3.8.6) on a workstation equipped with Apple M3 processors (8-core CPU, 10-core GPU) and 16 GB of RAM, running macOS Sonoma 14.6.1. Data preprocessing was performed in Python (v 3.12.3), and the MySQL database was managed via phpMyAdmin, ensuring structured and reproducible data handling. Subsequent statistical analyses were carried out in R (v 4.5.2), providing a consistent and transparent computational workflow across all stages of the study.

### The dataset

Between 2009 and 2015, 5,604 Italian women diagnosed with primary breast cancer at the Breast Unit of the National Cancer Institute of Naples (IRCCS “Fondazione G. Pascale”) were initially considered. After excluding 504 patients with incomplete data, the final cohort comprised 5,100 patients (age range: 18–96 years), including 3,841 who underwent breast‑conserving surgery (BCS, 75.3%) and 1,259 who underwent mastectomy (24.7%) (Fig. 4). Surgical procedures were verified through operative records, and classifications followed standardized institutional criteria. The study was retrospective, used anonymized data, and adhered to the Italian and European regulations in effect at the time, prior to GDPR implementation.

### The predictive model

Clinical prediction models (CPMs) and AI-based decision support systems have been increasingly applied in oncology to improve individualized care^34–36^. CPMs leverage patient‑ and disease‑specific data to provide personalized risk assessments and treatment recommendations, supporting clinicians in surgical and therapeutic decision‑making. For example, machine learning‑based clinical prediction models in oncology have been shown to generate individualized risk estimates that can inform prognosis and treatment strategy, highlighting both opportunities and challenges for clinical implementation^37,38^. BrCaM was developed to provide real-time surgical guidance by analyzing standardized patient data, including tumor characteristics, predicted disease progression, therapy response, and potential side effects. The final dataset was analyzed using supervised learning methods^39^, including:

- Boosting: AdaBoost, Real AdaBoost, MultiBoost AB
- Neural networks: Multilayer Perceptron (MLP)
- Decision trees: Logistic Model Tree (LMT), Random Forest
- Linear classifiers: Logistic Regression, SVM, SMO, SGD
- Instance-based: k-Nearest Neighbors (KNN)
- Probabilistic: Naive Bayes

All analyses were conducted following relevant guidelines.

### Validation protocol

Model validation employed 10-fold cross-validation on the 5,100-patient cohort, preserving class distributions. In each iteration, nine folds were used for training and one for testing. Preprocessing steps—duplicate removal, label harmonization, categorical encoding, and selective mode imputation—were applied consistently. Feature selection identified 21 informative prognostic variables. Performance metrics included overall accuracy, sensitivity and specificity for both surgical classes, precision, F1 score, and AUROC. Confusion-matrix–derived measures assessed classification errors and clinical relevance. This framework provided robust estimates of BrCaM’s discriminative performance and generalizability while minimizing overfitting. Nevertheless, the following potential threats: (i) Data Bias: The dataset is derived from a single institution, which could limit the generalizability of the model to other populations with different demographic or clinical characteristics; (ii) Model Overfitting: Although we used 10-fold cross-validation to assess model performance, there remains a potential risk of overfitting, especially given the relatively small number of patients undergoing mastectomy (24.7%); (iii) Clinical Variability: The model is based on retrospective data from a single surgical unit, which might not capture the full variability of surgical practices and patient preferences across different centers.

## Results

**Fig. 5:**
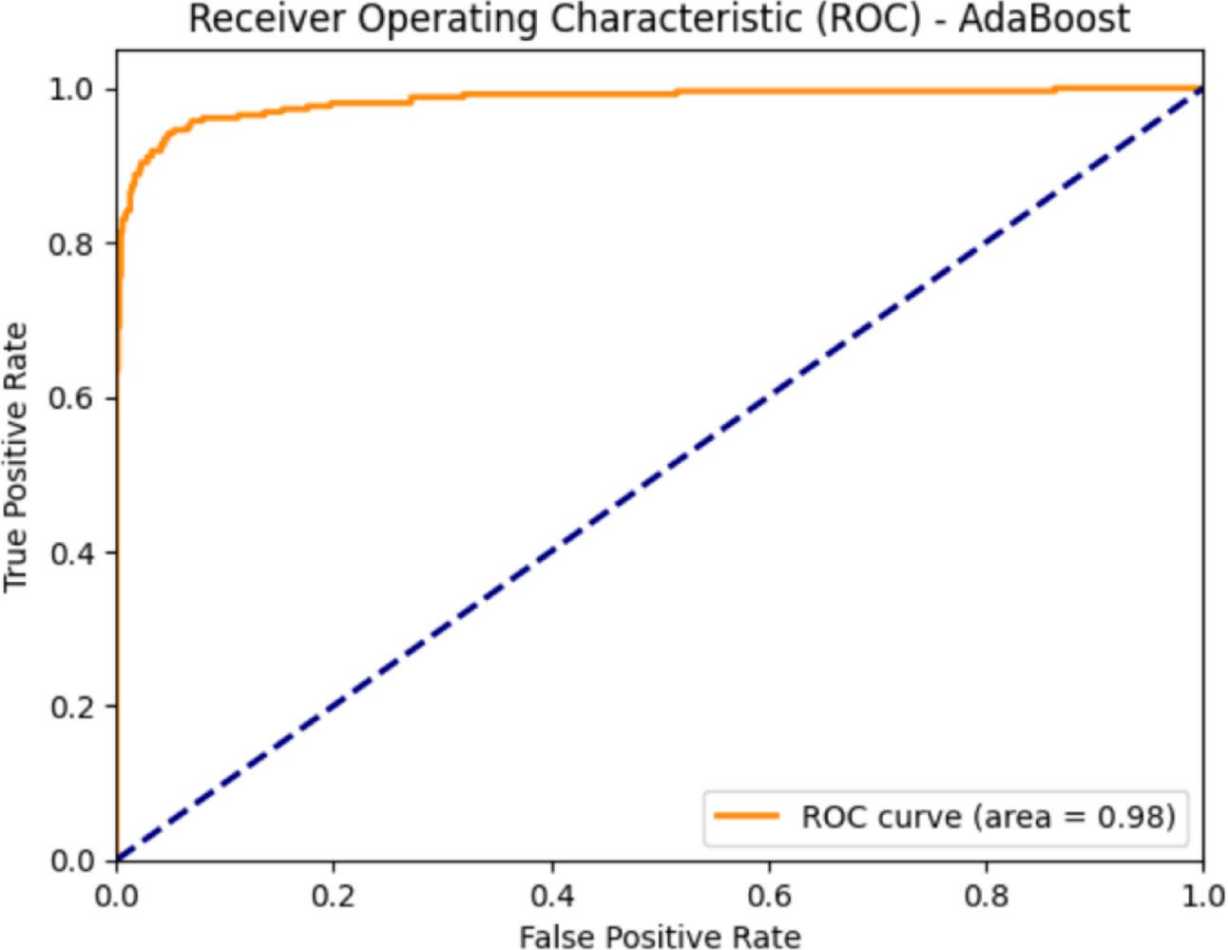
AdaBoost ROC curve.

**Fig. 6:**
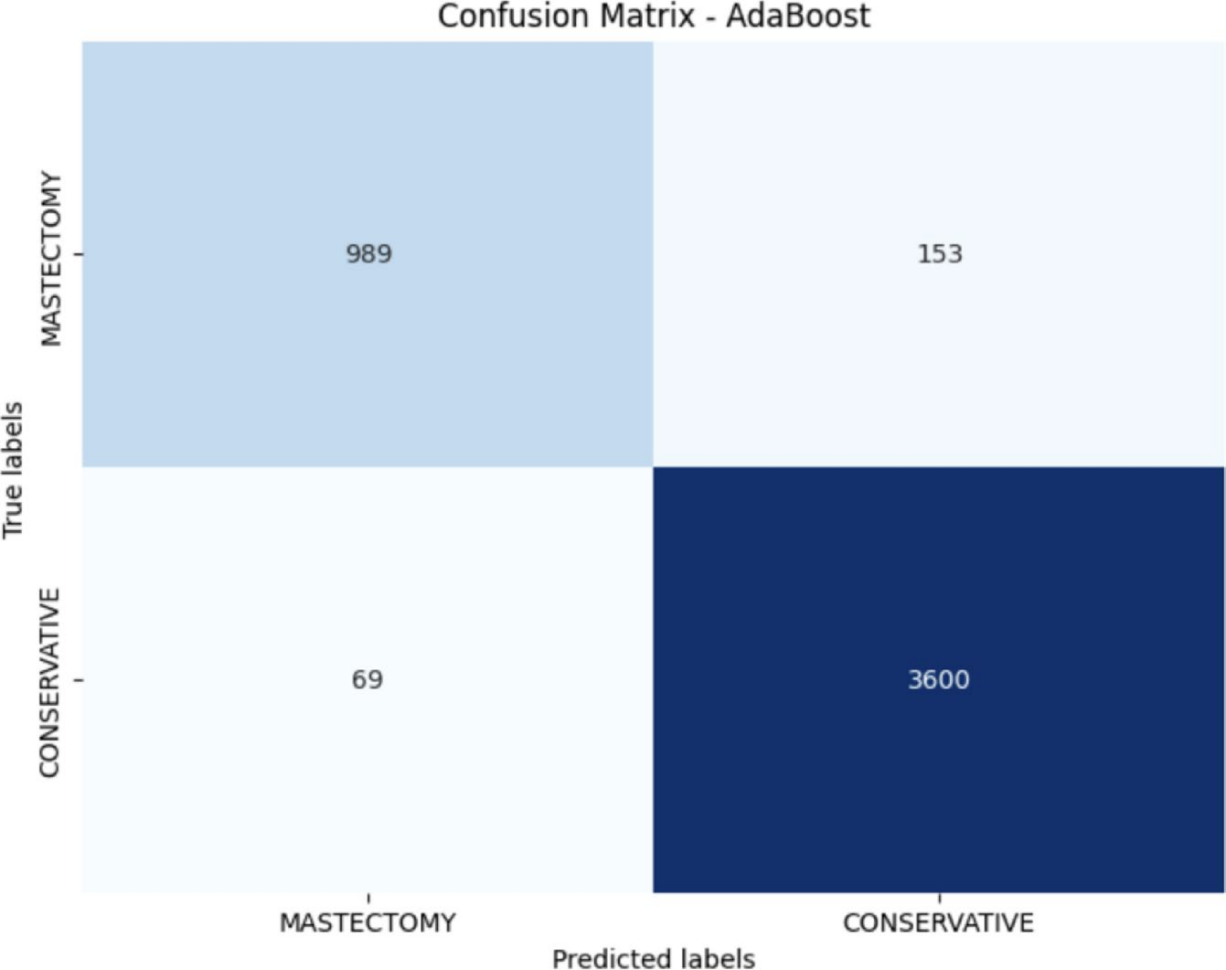
AdaBoost confusion matrix.

The developed computational pipeline provided a robust methodology for storing and analyzing breast cancer patient data to support personalized surgical planning. The final dataset included 5,100 patients, with 3,841 undergoing breast-conserving surgery (BCS) and 1,259 undergoing mastectomy, reflecting the real-world surgical distribution in our institution. A 10-fold cross-validation framework confirmed the model’s ability to accurately distinguish between conservative and ablative procedures. Among the evaluated machine learning algorithms, AdaBoost achieved the best overall performance. On the full cohort, the model correctly classified 3,772 out of 3,841 BCS cases and 1,190 out of 1,259 mastectomy cases, corresponding to an overall accuracy of 95.0%. The area under the receiver operating characteristic curve (AUROC) was 0.98, indicating excellent discriminative ability (Fig. 5). Confusion matrix analysis (Fig. 6) revealed 69 BCS cases misclassified as mastectomies (false positives) and 69 mastectomy cases misclassified as BCS (false negatives), totaling 138 misclassifications (2.7% error rate).

Model performance metrics are summarized in Table 1:

- *Breast-conserving surgery (BCS)*: sensitivity 96.2%, specificity 94.6%, F1-score 95.2%
- *Mastectomy*: sensitivity 94.5%, specificity 96.5%, F1-score 92.2%
- *Overall accuracy*: 95.0%
- *AUROC*: 0.98

**Table 1:** Results from 10-fold cross-validation technique, including performance metrics for breast-conserving surgery (BCS) and mastectomy prediction.

Classes	Algorithms	Accuracy	Precision	Recall	F1 score	TP-rate	FP-rate	RMSE	AUC-ROC
Ensemble	Ada Boost	0,95	0,95	0,95	0,95	0,95	0,11	0.19	0.98
	Multi Boost AB	0,94	0,94	0,94	0,94	0,94	0,12	0.19	0.96
	Real Ada Boost	0,92	0,92	0,92	0,92	0,92	0,14	0.19	0.94
Neural network	MLP	0,88	0,88	0,88	0,88	0,88	0,23	0.32	0.92
Decision tree	LMT	0,82	0,81	0,82	0,8	0,82	0,46	0.37	0.79
	Random forest	0,78	0,75	0,78	0,73	0,78	0,63	0.41	0.66
Linear	Logistic regression	0,82	0,81	0,82	0,8	0,82	0,45	0.37	0.79
	SMO	0,81	0,8	0,81	0,8	0,81	0,46	0.43	0.69
	SGD	0,81	0,8	0,8	0,8	0,81	0,46	0.43	0.68
	SVM	0,8	0,78	0,8	0,78	0,8	0,51	0.45	0.64
Instance-based	KNN	0,79	0,88	0,79	0,81	0,79	0,08	0.39	0.94
Probabilistic	Naive Bayes	0,79	0,77	0,79	0,76	0,79	0,56	0.39	0.74

These results suggest that the 21 selected prognostic factors provide high descriptive and predictive power, enabling reliable classification between surgical approaches. Misclassification impacts differ by direction: false negatives (predicting BCS when mastectomy was performed) may reflect unmeasured clinical risk factors or surgeon preference, potentially leading to suboptimal resection if followed blindly; false positives (predicting mastectomy when BCS was performed) represent overcautious predictions, which could result in unnecessary tissue removal. The model is intended as a decision-support tool, complementing clinical judgment rather than replacing it. Prediction confidence scores can further guide shared decision-making in borderline cases. Comparative analysis of alternative algorithms confirmed that AdaBoost outperformed Random Forest, Logistic Model Tree, Support Vector Machines, and Multilayer Perceptron in terms of accuracy, F1-score, and AUROC. These findings align with international surgical guidelines emphasizing individualized surgical planning based on tumor characteristics, patient anatomy, and personal preferences. Further to this, Our findings are consistent with recent studies demonstrating the effectiveness of machine learning and deep learning approaches for breast cancer diagnosis and clinical decision support^40–42^. Prior work has shown that advanced ML and DL models can achieve high discriminative performance by capturing complex, non-linear relationships within heterogeneous clinical datasets, thereby supporting more informed and personalized clinical decisions. Therefore, the proposed predictive framework demonstrates the potential to reduce uncertainty in surgical decision-making, improve preoperative planning, and support patient-centered care.

## Discussion

This study presents BrCaM, an artificial intelligence–based clinical decision-support model designed to assist pre-operative surgical planning in breast cancer by predicting the likelihood of breast-conserving surgery versus mastectomy. Using a large, homogeneous single-institution cohort and a curated set of pre-operative clinical, anatomical, and diagnostic variables, the model demonstrated strong discriminative performance, supporting the feasibility of data-driven approaches for surgical decision support in routine clinical practice. Given the retrospective design of the study, BrCaM does not provide de novo surgical recommendations but rather models and validates the clinical criteria that guided surgical decisions in routine clinical practice. A key strength of BrCaM is its focus on a clinically underexplored feature i.e. pre-operative surgical decision-making. While most machine learning applications in breast cancer emphasize diagnosis, prognosis, or survival prediction, relatively few address the selection of surgical approach. By leveraging routinely available variables—such as tumor focality, lymph node status, imaging assessments, and anatomical constraints—BrCaM captures clinically meaningful patterns that influence real-world surgical decisions. The observed performance across multiple metrics suggests that these factors provide sufficient predictive signal to support individualized surgical planning. Importantly, the model was trained on data reflecting actual clinical practice, incorporating both objective criteria and surgeon-guided decision patterns within a standardized institutional framework. Although surgical decision-making may include subjective elements, this variability likely enhances real-world applicability by allowing the model to learn from authentic clinical behavior rather than theoretical rules. Accordingly, BrCaM is intended to function as a decision-support tool that complements, rather than replaces, multidisciplinary clinical judgment. We also acknowledge certain limitations in our study such as the retrospective, single-center design may limit generalizability to settings with different patient populations or surgical practices, underscoring the need for independent external validation. Additionally, certain relevant factors - including detailed lifestyle variables, psychosocial considerations, and genetic risk markers - were not included due to data availability constraints. Accordingly, the observed predictive performance should be interpreted as confirmation of the alignment between measurable clinical factors and real-world surgical choices, rather than as evidence for prescriptive clinical use at this stage. Future development will focus on expanding the feature space to incorporate genomic and molecular data, such as BRCA1/2 mutation status, multigene assay scores, and molecular subtypes, as well as radiomics features extracted from mammography, ultrasound, and MRI using deep learning. Integration of multi-omics data has the potential to enable more precise surgical planning by accounting for tumor biology alongside anatomical and clinical factors. However, this will require prospective data collection and evolution of the model architecture toward multimodal frameworks, supported by eXplainable artificial intelligence techniques to ensure transparency and clinical trust. Our findings support individualized surgical decision‑making that considers tumour characteristics, patient anatomy, and personal preferences, in line with current international guidelines, including the NCCN Clinical Practice Guidelines in Oncology – Breast Cancer and the ESMO Clinical Practice Guidelines for Early Breast Cancer^43,44^.

### Clinical implementation and usability considerations

To facilitate clinical adoption, we prioritized usability alongside model performance, recognizing that high accuracy alone is insufficient for safe deployment. A clinician-centered assessment is essential for effective implementation. At its current stage, BrCaM represents a proof-of-concept prediction model, achieving 95% accuracy in retrospective internal validation against historical surgical decisions. Developed in close collaboration with breast cancer surgeons to ensure predictor selection aligns with established clinical criteria, the model follows TRIPOD-AI guidance for transparent reporting. Expert feedback confirmed that the selected prognostic factors reflect the parameters routinely considered during surgical planning. In accordance with CONSORT-AI standards, the next phase focuses on prospective human-factors evaluation, specifically:

1. Assessing interface design and workflow compatibility;
2. Evaluating interpretability and trust via think-aloud and cognitive walkthrough methodologies;
3. Measuring impacts on decision-making efficiency;
4. Identifying systemic barriers to adoption.

Full integration into routine practice remains contingent upon successful usability testing, regulatory review, and prospective validation, ensuring the responsible translation of AI support into the surgical suite.

In summary, BrCaM represents a significant step toward data-driven surgical decision support. By demonstrating strong performance using routinely available pre-operative data, this study highlights the potential of machine learning to reduce uncertainty and complement clinical expertise in personalized surgical planning.

## Conclusion

In this study, we developed and trained BrCaM (Breast Cancer Surgical Planning), an AI-based clinical prediction model designed to model pre-operative surgical decision-making patterns in breast cancer. Using a dataset of 5,100 patients and 21 clinically relevant prognostic factors, the model achieved a predictive accuracy of 95%, demonstrating strong discriminative performance between breast-conserving surgery (BCS) and mastectomy. Given the retrospective nature of the study, BrCaM should not be interpreted as a prescriptive tool for recommending surgical approaches. Rather, the model captures well-established clinical criteria already guiding real-world surgical decisions, thereby confirming the appropriateness and consistency of current patient–surgeon decision-making processes. Several limitations should be acknowledged, including the influence of surgeon experience and patient preferences on surgical choice, as well as the single-center design. Importantly, training the model on real-world decisions may enhance its ecological validity by reflecting routine clinical practice rather than idealized guideline-based scenarios. Future work will focus on external and prospective validation, comparing model predictions with surgical decisions made in real time, as well as on integrating explainable AI techniques and multi-omics data. Overall, BrCaM provides a robust data-driven framework for understanding surgical decision-making in breast cancer and establishes the basis for future development of clinically deployable decision-support tools.

## Supplementary Information

Below is the link to the electronic supplementary material.

Supplementary Material 1

Supplementary Material 2

Supplementary Material 3

Supplementary Material 4

Supplementary Material 5

## Data Availability

The data that support the findings of this study are available from the corresponding author or at the following URL: [https://github.com/Lucasilvestri11/An\_AI\_predictive\_model\_for\_surgical\_planning\_in\_breast\_cancer\_on\_women\_with\_unhealthy\_eating\_habits].
