# Supplementary material for: BrCaM an artificial intelligence model for surgical decision making in breast cancer
Source: Sci Rep. 2026 Mar 16;16:13598. doi: 10.1038/s41598-026-43281-6 (PMC13121618; doi:10.1038/s41598-026-43281-6)
Supplement: Supplementary file 1 — Supplementary Material 1 [file 41598_2026_43281_MOESM1_ESM.pdf]

regione campania
aslnapoli3sud

REGIONE CAMPANIA
AZIENDA SANITARIA LOCALE NAPOLI 3 SUD
Sede legale- Torre del Greco (NA) Via Marconi,66
Pec: protocollo@pec.aslnapoli3sud.it

BREAST UNIT AZIENDALE
Centro di Senologia Multidisciplinare
Direttore UOC **Dr. M D'Aiuto**
Email: breastunit@aslnapoli3sud.it
Tel.: +39 0815352925

P.O. Boscotrecase
"S. Anna e S.S. Madonna della Neve"

The study was conducted using historical medical records (2009–2015) that were fully anonymized prior to analysis. According to institutional policy and applicable regulations, no formal ethics approval was required for the use of data as they were acquiredbefore the GDPR - (UE) n. 2016/679.

15 December 2025

Signature

Massimiliano D'Aiuto
