# Supplementary material for: BrCaM an artificial intelligence model for surgical decision making in breast cancer
Source: Sci Rep. 2026 Mar 16;16:13598. doi: 10.1038/s41598-026-43281-6 (PMC13121618; doi:10.1038/s41598-026-43281-6)
Supplement: Supplementary file 4 — Supplementary Material 4 [file 41598_2026_43281_MOESM4_ESM.docx]

**Performance Calculations for *BrCaSP* Model**

**1. Confusion Matrix**

| **True / Predicted** | **BCS (Conservative)** | **Mastectomy** | **Total** |
| --- | --- | --- | --- |
| BCS (Actual) | 3,708 (TN) | 133 (FP) | 3,841 |
| Mastectomy | 69 (FN) | 1,190 (TP) | 1,259 |
| **Total** | 3,777 | 1,323 | 5,100 |

*Abbreviations:* TN = True Negative, TP = True Positive, FP = False Positive, FN = False Negative.

**2. Performance Metrics**

| **Metric** | **Formula** | **Value** |
| --- | --- | --- |
| **Accuracy** | $\left( TP+TN)/(TP+TN+FP+FN \right)$ | 96.0% |
| **Sensitivity (Recall, Mastectomy)** | $TP/(TP+FN)$ | 94.5% |
| **Specificity (BCS)** | $TN/(TN+FP)$ | 96.5% |
| **Precision (Mastectomy)** | $TP/(TP+FP)$ | 89.9% |
| **F1 Score (Mastectomy)** | $2\times\frac{Precision\times Recall}{Precision+Recall}$ | 92.2% |
| **Error Rate** | $(FP+FN)/Total$ | 4.0% |
| **AUROC** | — | 0.98 |
|  |  |  |

**3. Step-by-Step Calculations**

1. **Accuracy:**

$$\text{Accuracy}=\frac{1,190+3,708}{5,100}=0.960\text{ }(96.0\%)$$

2. **Sensitivity (Recall, Mastectomy):**

$$\text{Sensitivity}=\frac{1,190}{1,190+69}\approx0.945\text{ }(94.5\%)$$

3. **Specificity (BCS):**

$$\text{Specificity}=\frac{3,708}{3,708+133}\approx0.965\text{ }(96.5\%)$$

4. **Precision (Mastectomy):**

$$\text{Precision}=\frac{1,190}{1,190+133}\approx0.899\text{ }(89.9\%)$$

5. **F1 Score (Mastectomy):**

$$F1=2\times\frac{0.899\times0.945}{0.899+0.945}\approx0.922\text{ }(92.2\%)$$

6. **Error Rate:**

$$\text{Error}\text{ Rate}=\frac{133+69}{5,100}\approx0.040\text{ }(4.0\%)$$

7. **AUROC:** Aggregated over 10-fold cross-validation: 0.98.
