# Supplementary material for: BrCaM an artificial intelligence model for surgical decision making in breast cancer
Source: Sci Rep. 2026 Mar 16;16:13598. doi: 10.1038/s41598-026-43281-6 (PMC13121618; doi:10.1038/s41598-026-43281-6)
Supplement: Supplementary file 5 — Supplementary Material 5 [file 41598_2026_43281_MOESM5_ESM.docx]

| **FEATURES NAME** | **AVERAGE MERIT** | **AVERAGE RANK** | **MATCH** |
| --- | --- | --- | --- |
| ricostruzione | *0.513 ± 0.005* | *1 ± 0* | *✓* |
| biRadsClinico | *0.198 ± 0.004* | *2 ± 0* | *✓* |
| familiarità_carcinoma_ovarico | *0.154 ± 0.002* | *3 ± 0* | *✓* |
| focalità | *0.143 ± 0.003* | *4 ± 0* | *✓* |
| rapporto_cuteSX | *0.029 ± 0.001* | *5 ± 0* | *✓* |
| rapporto_cuteDX | *0.024 ± 0.001* | *6 ± 0* | *✓* |
| rapporto_areola_capezzoloDX | *0.018 ± 0.001* | *7.8 ± 0.87* | *✓* |
| rapporto_areola_capezzoloSX | *0.018 ± 0.001* | *8.1 ± 0.94* | *✓* |
| struttura ghiandolare | *0.018 ± 0.001* | *8.4 ± 0.92* | *✓* |
| stato_linfonodaleSX | *0.016 ± 0.001* | *9.7 ± 0.64* | *✓* |
| citologia_codifica | *0.012 ± 0.001* | *11.8 ± 0.98* | *✓* |
| stato_linfonodaleDX | *0.012 ± 0.001* | *12.1 ± 0.7* | *✓* |
| fumo | *0.012 ± 0.001* | *12.8 ± 1.17* | *✓* |
| età | *0.011 ± 0.001* | *13.7 ± 1.42* | *✓* |
| gravidanza | *0.010 ± 0.001* | *15.1 ± 0.7* | *✓* |
| citologia | *0.009 ± 0.001* | *15.5 ± 0.5* | *X* |
| casi_seno_famiglia | *0.005 ± 0.001* | *17.2 ± 0.4* | *X* |
| menopausa | *0.005 ± 0.001* | *17.8 ± 0.4* | *X* |
| lato_intervento | *0.001 ± 0* | *19.5 ± 0.81* | *X* |
| peso | *0.001± 0* | *20.1 ± 0.54* | *X* |
| altezza | *0.001± 0* | *20.4 ± 0.8* | *X* |
